# New Appliances and Things Medical

**Published:** 1906-07-14

**Authors:** 


### NEW APPLIANCES AND THINGS MEDICAL.

[We shall be glad to receive, at our Office, 28 & 29 Southampton Street, Strand, London, W.C., from the manufacturers, specimens of all new preparations and appliances which may be brought out from time to time.]

### BLACK AND WHITE SCOTCH WHISKY (HOUSE OF COMMONS WHISKY.)

(Messrs. James Buchanan and Co., Ltd., Glasgow and London).

Chemical analysis and expert tasting of this whisky carried out by us show that Black and White is a Scotch whisky of the very highest character. It has a sufficiency of body, combined with elegance and flavour, and what is, perhaps, of even greater importance, it is thoroughly mature. The figures obtained by analysis (the whisky being purchased by us on the open market) were: Alcohol, 46.7 per cent. by vol.; Extractives, 0.17 per cent.; Ash, 0.01 per cent. The following figures represent parts per 100,000 of absolute alcohol: Total acid, 58; non-volatile acid, 16; volatile acid, 42; compound ethers, 57; total aldehydes, 20; furfural, 3.2; higher alcohols (by the Allen-Marquardt process), 134; ditto by the colorimetric process, 242; co-efficient of "impurities," 380.2. Although we have not much faith in chemical "standards," it is yet of interest to note that Black and White is fully up to the chemical standard demanded by a public analyst in a recent case for genuine Scotch whisky. We are not surprised that this whisky has attained a world-wide popularity, inasmuch as it is only blending of the most expert character combined with the use of excellent and mature materials which could produce a beverage which strikes, as does Black and White, such a happy medium between the excessively "fat" and heavy Scotch whiskies on the one hand, and the ultra thin types on the other hand. As a beverage and general dietetic agent, we know of no better Scotch whisky. It can be safely recommended by any practitioner to a patient who requires a stimulant, the action of which it is desired shall be more sustained than that of brandy. It is also within easier reach of the purse and more readily obtained than suitable brandy.

